# SPARC‐related modular calcium‐binding proteins (SMOC1 and 2) as a modulator of amyloid pathology in Alzheimer’s Disease

**DOI:** 10.1002/alz.091697

**Published:** 2025-01-03

**Authors:** Kazi Farhana Afroz, Yona Levites, Danny Ryu, Yong Ran, Joshna Dharmendrabhai Gadhavi, Xuefei Liu, Nicholas T Seyfried, Todd Golde

**Affiliations:** ^1^ Emory University, Atlanta, GA USA; ^2^ Emory University Center for Neurodegenerative Disease, Atlanta, GA USA

## Abstract

**Background:**

Recently, a highly significant brain proteome divergent modules change between Alzheimer’s disease (AD) and CRND8 APP_695NL/F_ transgenic mice has been found. The M42 module is the module in human AD most highly correlated with amyloid and tau pathologies and cognitive decline. Among all proteins in this module, the (SPARC‐related modular calcium‐binding protein 1) SMOC1 is emerging as a robust biomarker of amyloid deposition in CSF. It increases in CSF in parallel with the Ab42:40 ratios’ decrease. SMOC1 is also markedly increased in the late‐onset AD CSF, suggesting its role as a robust biomarker. Our preliminary data have shown that i) recombinant SMOC1 inhibits Aβ aggregation *in vitro* and ii) both SMOC1 and its homolog SMOC2 bind amyloid structures. Furthermore, SMOC1 and 2 appear to be components of a subset of senile plaques and CAA.

**Method:**

We hypothesized that Smoc1 and Smoc2 overexpression will inhibit amyloid deposition and even promote clearance of aggregated Aβ. To test the hypothesis, we cloned both genes into AAV and injected newborn CRND8 amice with rAAV2/8‐Smoc1‐Flag and rAAV2/8‐Smoc2‐Flag, aged them and assessed amyloid deposition in the brain by immunohistochemical and biochemical methods.

**Result:**

Immunohistochemical analysis showed that Smoc1 overexpression resulted in significantly lower amyloid plaque count and SDS‐soluble Aβ1‐42 and Aβ1‐40 levels in the brain, as compared to non‐injected controls. Soluble Aβ1‐42 and Aβ1‐40 levels were not changed as a result of Smoc1 overexpression. Staining with astrocyte‐specific glial fibrillary acid protein (GFAP) and microglia‐specific ionized calcium‐binding molecule‐1 (IBA‐1) antibodies showed that Smoc1 overexpression did not affect astrocytosis, but significantly increased the activated microglia cell counts. Additionally, we are currently examining the effects of Smoc1 and Smoc2 overexpression on pre‐existing plaques in aged CRND8 mice.

**Conclusion:**

Our data suggest that Smoc1, in addition to being an early and sensitive fluid biomarker, is acting as an inhibitor of amyloid formation and can potentially lead to a novel therapeutic approach. Although Smoc1 and Smoc2 prevent Aβ aggregation, the specific mechanism of this action is yet to be identified. Our findings will pave the road to establishing SMOC proteins as a potential therapy for AD.

